# Impact of Voxel Grid Size and Statistical Uncertainty on Surface Depth Dose Via Various Planning Techniques and Immobilization Devices Using Monte Carlo Algorithm

**DOI:** 10.1177/15330338261425904

**Published:** 2026-03-20

**Authors:** Srinivas Challapalli, Anupam Choudhary, Jyothi Nagesh, Shambhavi C, Shirley Lewis, Umesh Velu, Jayashree NP, Ankita Mehta, Manoj Belwal, Dilson Lobo, Sarath S. Nair

**Affiliations:** 1 Department of Radiation Oncology, 76798 Kasturba Medical College Mangalore, Manipal Academy of Higher Education, Manipal, Karnataka India; 2 Department of Urology, Kasturba Medical College, Manipal Academy of Higher Education, Manipal, India; 3 Department of Radiation Oncology, Kasturba Medical College, Manipal Academy of Higher Education, Manipal, India; 4 Medical Physics programme, 76799 Manipal College of Health Professions, Manipal Academy of Higher Education, Manipal, Karnataka, India; 5 Welcomgroup Graduate School of Hotel Administration, 177107 Manipal Academy of Higher Education, Manipal, Karnataka, India

**Keywords:** dose voxel size, statistical uncertainty, monte carlo, intensity modulated radiation therapy, volumetric arc therapy (VMAT)

## Abstract

**Introduction:**

This study aims to analyze the impact of the surface dose at depth (1-5mm) in different planning techniques and immobilization devices by varying the dose-voxel size (DVS) and statistical uncertainty (SU) using Monte Carlo (MC) algorithm.

**Methods:**

Three Sets of computed tomography (CT) images were taken from an in-house developed chest phantom, which included an open phantom, a vaclok and a thermoplastic mask. The image sets were pushed to the Monaco planning station for registration and contouring. Six beams of 6 MV photon energy are used to plan an Intensity modulated radiotherapy (IMRT) technique, and a half arc beam is used for Volumetric Modulated arc therapy (VMAT). In each plan, recalculation is performed by changing only the grid size from 1 mm to 8 mm and the statistical uncertainty from 1% to 5% from the parameter control window, keeping the other dose constraints the same. A total of 240 plans were performed for all three image sets together for both the IMRT and VMAT techniques, and the dose at depth was compared and statistically analyzed via the Kruskal–Walli's test.

**Results:**

The homogeneity index (HI), conformity index (CI), and V95% target are increased in both IMRT and VMAT, whereas the SU and DVS are reduced.

**Conclusion:**

Higher statistical uncertainty and grid size significantly reduced dose calculation time, independent of technique or device. surface dose at depth (1-5mm) decreased with increasing grid size and increased with lower statistical uncertainty. IMRT consistently showed higher skin doses than VMAT across all devices, with vac-lock immobilization yielding the highest surface dose in both techniques. These findings show that the surface dose is influenced by beam selection, parameter settings, and the optimization time allocated during treatment planning.

## Introduction

Cancer is one of the major causes of morbidity and mortality worldwide. This is commonly known fact that the use of adjuvant radiation therapy plays a crucial role in treatment as part of the management of nonmetastatic early and locally advanced cancer^.^^1–3^ Accurate dose calculation and delivery in radiotherapy are essential to maximize tumor control while minimizing toxicity to surrounding healthy tissues. Among the critical factors influencing treatment outcomes, skin dose remains a particular concern, as excessive radiation to the surface can lead to acute and late skin reactions that compromise patient comfort, compliance, and overall treatment quality. It is generally established that patient skin dosage is significantly affected by immobilization devices^4–6^ along with other machine parameters, where skin toxicity is often considered a dose-limiting factor in radiation therapy between cosmesis and radiation outcomes.^7–9^ Hence, it is clinically crucial to limit the skin dose accurately during treatment planning. With advanced treatment modalities such as volumetric modulated arc therapy (VMAT) and intensity-modulated radiotherapy (IMRT), this toxicity can be limited. To generate these types of intensity-modulated plans, a more sophisticated dose calculation algorithm is necessary for the treatment planning system (TPS). They play a crucial role in the dose calculation for generating a quality plan and preventing the mistreatment of radiation delivery to patients. TPS uses several mathematical formulations and algorithms for quick and precise dose calculations. This algorithm works on the configured input radiation beam data measured during the time of machine commissioning. There are many TPS available that can be used to develop VMAT and IMRT plans, each utilizing their own dose calculation algorithm, such as superposition, anisotropic analytical algorithms, collapsed cones, Acuros XB, and Monte Carlo (MC) methods. This MC algorithm, which is a statistical simulation-based calculation, is considered the most accurate and precise dose computation method for treatment planning.^10,11^ While the MC-based treatment planning approach is utilized, certain parameters need to be selected for the optimum dose calculation, such as the dose volume size (DVS) and the level of statistical uncertainty (SU) or statistical noise. These parameters play a significant role in determining both the precision of the displayed dose distribution and the time required to complete the calculation. In the context of the Monaco planning station (version 5.11), these parameters are critical for the accuracy and efficiency of the MC simulation. The DVS refers to the size of the grid used to discretize the patient's anatomy for dose calculations, which is adjustable and ranges from 1 mm to 8 mm at our Monaco planning station, allowing planners to balance the calculation speed and accuracy. Moreover, the SU refers to the inherent random fluctuations in the MC simulation due to the probabilistic nature of the algorithm. These fluctuations can be mitigated by increasing the number of simulated particles in the MC calculation. The Monaco planning station provides the flexibility to set SU values between 1% and 5%, enabling the optimization of precision and computation time at our planning station. Thus, in MC-based calculations, the dose distribution region is divided into voxels of equal volume, and interpolation of this voxel helps in total dose computation, whereas the SU value controls the statistical noise within the dose calculation.^12^ Statistical noise is always inversely proportional to the SU value of the calculation parameters. Studies have been performed previously to understand the effects of SUs on the MC algorithm^13–15^; however, these studies have focused on only one of the parameters, and most of them have focused mainly on the VMAT planning technique. To the best of our knowledge, no precise data is available regarding the surface or skin dose with varying SU and DVS in different planning techniques and on different immobilisation devices.

Surface dose is influenced not only by beam configuration and treatment technique, but also by immobilization devices, planning system parameters, and uncertainties inherent to dose calculation algorithms. Majorities of the investigations have often addressed these variables in isolation, with limited work has explored their combined impact. To address this gap, in our study a locally constructed elliptical phantom was developed using tissue-equivalent materials (acrylic, cork, Teflon) to simulate anatomical structures, including lung, spinal cord, and chest wall. This phantom enabled a high degree of experimental control, allowing for simultaneous evaluation of device effects, planning techniques, and algorithmic variations on surface dose. In this study, the influence of two widely used immobilization devices vacuum cushion (Vaclok) and thermoplastic mask were assessed along with two advanced treatment techniques, VMAT and IMRT. The analysis further incorporated variations in DVS and SU parameters of the MC algorithm in the Monaco treatment planning system. This multi-factorial approach provides a comprehensive perspective on how immobilization, planning technique, and calculation parameter settings influence the skin dose. The novelty of this work lies in its multi-dimensional evaluation of clinical and technical factors that together shape surface dose accuracy. By integrating device considerations with planning and algorithmic parameters, this study offers valuable insights into optimizing radiotherapy planning to reduce skin toxicity and improve treatment precision. These findings hold direct implications for clinical decision-making, particularly in selecting appropriate immobilization, Treatment technique and the planning parameters that balance treatment efficacy with patient safety.

## Materials and Methods

**Figure 1.**
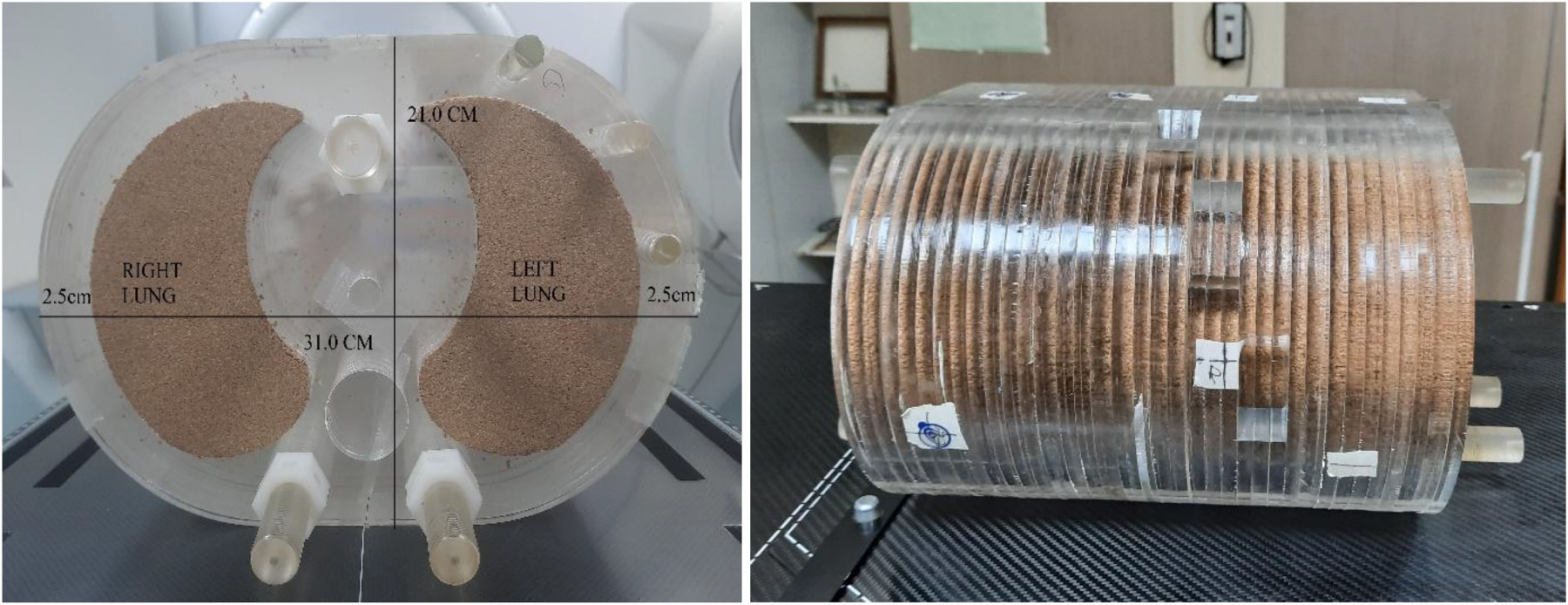
Axial and lateral view of in-house phantom used.

The study was conducted using a locally constructed elliptical phantom 21 cm in height and 31 cm in width, which consists of thirty acrylic plates with a density of 1.04 g/cc.^16^ To mimic the lung, cork material with a density of 0.28 gm/cc was used, and for the spinal cord, Teflon material with a density of 1.65 g/cc was used, as shown in Figure 1 below.

**Figure 2.**
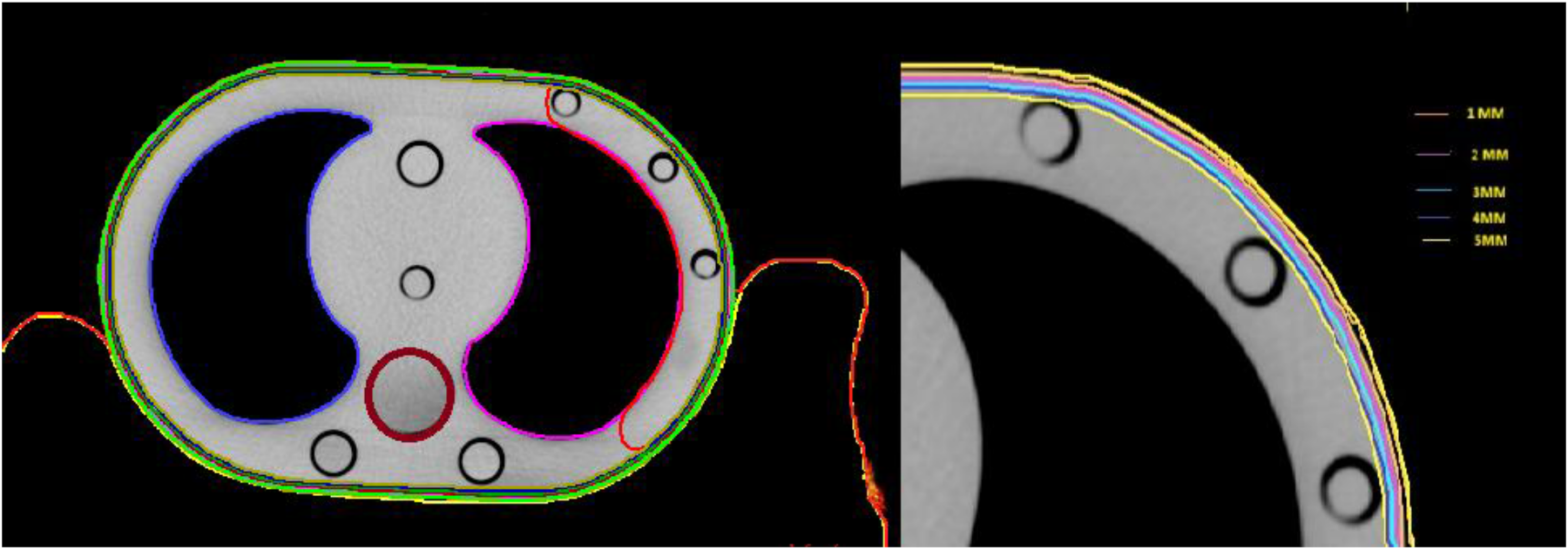
Organ-at-risk delineation with skin marks at 1–5 mm depth.

Different types of immobilization devices are used in the radiotherapy department for the precise treatment of cancer tissue, which helps arrest the movement of the patient and helps spare normal tissue to some extent. In our department, the most used immobilization devices are thermoplastic masks (Orfit) and blue bag vaclok cushion. Three sets of images were taken via the elliptical chest phantom by the Philips brilliance 16 big bore computerized tomography (CT) machine, one with thermoplastic immobilization, another with a blue bag vaclok blue, and the last one is an open image set (without any immobilization device). The acquired 3 mm slice images of all three sets were later transferred to the Monaco planning station (Elekta Ltd, Stockholm, Sweden) for further registration after the CT-to-ED conversion. Contouring, such as the right and left lungs, spinal cord, Ptv and skin up to 1–5 mm, was performed in all images sets along with the thermoplastic mask and vaclok immobilization devices per the contouring protocol in the Monaco contouring station (shown in Fig. 2).

**Figure 3.**
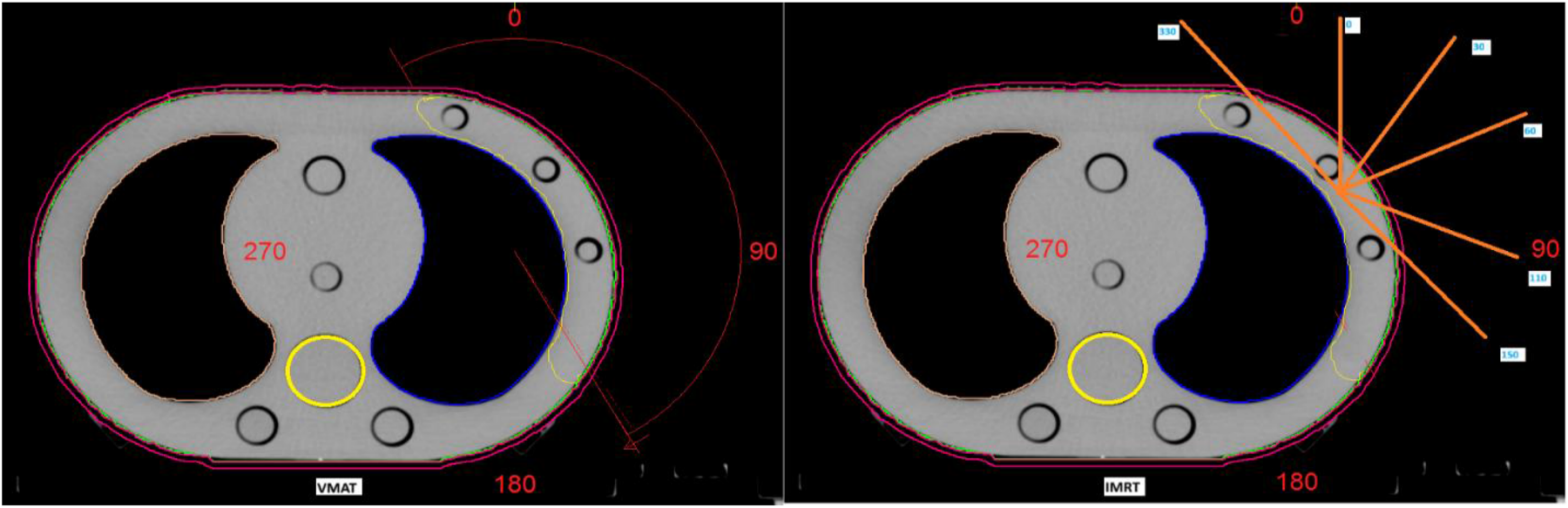
VMAT and IMRT treatment plans generated using the elliptical phantom.

Six beams (Gantry of 330°, 0°, 30°, 60°, 110°, and 150°) with photon energies of 6 mv were used for the IMRT technique, and a half arc VMAT plan starting from 330° to 150° was used for each image set for a dose of 42.5 Gy in 16 fractions via the MC algorithm (Fig 3).

The constraint parameters were given such that the minimum target coverage of 95%, as per the institutional protocol, was followed. Each plan had been previously optimized and approved by an experienced oncologist prior to performing the dose recalculations. For the dose calculations, a Hewlett-Packard Z820 workstation with 128 GB of RAM, an Intel Xeon CPU E5-2695 @ 2.40 GHz (2-Processor), and a 64-bit operating system were used. For each grid spacing (from 1 mm to 8 mm), dose recalculations were performed along with varying SU (1% to 5%). A total of 240 optimization plans (120 IMRT plans and 120 VMAT plans) were performed for all three image sets together for both the IMRT and VMAT planning techniques. These plans covered the entire range of SU values (1%-5%) and DVS (1 mm-8 mm) sizes. To assess the outcomes, data were extracted from the DVH for each DVS & SU for each plan for all three sets, the maximum dose values presented in Table 1 were obtained from the DVH as the dose at a single point within the specified skin thickness (1-5 mm), The collected data were meticulously evaluated, offering insights into how different planning parameters impact the dose distribution on the skin layers.

**Table 1.** Surface Depth Dose from 1mm to 5mm (Gy) for Varying DVS (1mm −8mm) and SU (1%-5%) for VMAT & IMRT Treatment Planning a) With Vaclok, b) With Mould, c) Without Immobilisation Device (DVS: Dose Volume Size, SU: Statistical Uncertainty, VMAT: Volumetric Modulated Arc Therapy, IMRT: Intensity Modulated Radiation Therapy).

VMAT	MOULD-DVS 1mm Surface thickness (mm)					MOULD-DVS 2mm Surface thickness (mm)					MOULD-DVS 3mm Surface thickness (mm)					MOULD-DVS 4mm Surface thickness (mm)				
**SU%**	**1**	**2**	**3**	**4**	**5**	**1**	**2**	**3**	**4**	**5**	**1**	**2**	**3**	**4**	**5**	**1**	**2**	**3**	**4**	**5**
**1%**	41.83	43.77	45.21	46.18	47.19	42.04	43.68	45.46	46.03	47.06	41.45	42.78	44.31	45.42	46.25	41.61	42.56	44.03	44.61	45.45
**2%**	44.26	45.22	46.07	47.73	48.19	43.14	44.61	46.01	46.02	47.98	43.25	43.95	46.01	46.71	47.62	41.88	43.29	44.57	45.82	46.44
**3%**	45.19	46.35	47.57	48.66	49.65	45.69	46.38	46.58	48.08	48.08	43.95	44.72	45.51	46.49	47.73	43.32	43.88	46.65	46.65	46.85
**4%**	45.19	46.35	47.57	48.66	49.65	45.69	46.38	46.58	48.08	48.08	43.95	44.72	45.51	46.49	47.73	43.32	43.88	46.65	46.65	46.85
**5%**	45.19	46.35	47.57	48.66	49.65	45.69	46.38	46.58	48.08	48.08	43.95	44.72	45.51	46.49	47.73	43.32	43.88	46.65	46.65	46.85
	MOULD-DVS 5mm Surface thickness (mm)					MOULD-DVS 6mm Surface thickness (mm)					MOULD-DVS 7mm Surface thickness (mm)					MOULD-DVS 8mm Surface thickness (mm)				
**SU%**	**1**	**2**	**3**	**4**	**5**	**1**	**2**	**3**	**4**	**5**	**1**	**2**	**3**	**4**	**5**	**1**	**2**	**3**	**4**	**5**
**1%**	41.59	42.36	43.96	44.85	45.84	40.79	42.08	43.04	44.05	45.11	40.23	42.02	43.65	44.16	44.88	40.14	41.85	43.29	43.8	44.06
**2%**	42.55	43.55	45.26	46.21	47.17	42.67	43.06	44.37	44.82	46.29	41.14	43.2	44.14	44.82	46.16	41.33	42.5	43.8	44.8	45.79
**3%**	42.24	43.35	45.07	46.28	47.21	43.43	43.91	45.45	46.45	47.23	41.78	44.2	44.53	45.15	46.54	43.37	43.38	43.92	44.45	45.39
**4%**	42.24	43.35	45.07	46.28	47.21	43.43	43.91	45.45	46.45	47.23	41.78	44.2	44.53	45.15	46.54	43.37	43.38	43.92	44.45	45.39
**5%**	42.24	43.35	45.07	46.28	47.21	43.43	43.91	45.45	46.45	47.23	41.78	44.2	44.53	45.15	46.54	43.37	43.38	43.92	44.45	45.39

**Table 1A.** Surface Depth Dose for VMAT and IMRT Using Mould Immobilisation Device.

IMRT	MOULD-DVS 1mm Surface thickness (mm)					MOULD-DVS 2mm Surface thickness (mm)					MOULD-DVS 3mm Surface thickness (mm)					MOULD-DVS 4mm Surface thickness (mm)				
SU%	1	2	3	4	5	1	2	3	4	5	1	2	3	4	5	1	2	3	4	5
1%	44.89	45.9	46.69	47.3	47.47	44.37	45.7	46.38	46.88	46.99	43.42	44.93	46.08	46.54	46.83	43.41	44.59	45.47	45.82	46.83
2%	48.48	48.48	49.58	49.81	49.81	47.26	47.29	49.68	49.68	49.68	45.57	47.56	48.07	48.7	48.7	46.23	46.23	46.99	47.1	48.33
3%	54.45	53.8	53.8	53.81	54.59	51.17	53.27	53.27	53.27	53.27	51.36	52.02	52.02	52.02	52.02	48.6	50.84	50.99	51.39	51.78
4%	59.08	59.08	59.08	59.08	59.08	57.0	57.02	63.25	63.25	63.25	53.29	53.29	53.69	53.69	53.69	53.62	53.67	53.67	53.67	54.55
5%	60.59	61.02	61.02	61.02	61.02	56.82	57.47	57.47	59.07	59.07	61.97	63.2	63.2	63.2	63.2	56.33	61.14	61.14	61.14	61.14
	MOULD-DVS 5mm Surface thickness (mm)					MOULD-DVS 6mm Surface thickness (mm)					MOULD-DVS 7mm Surface thickness (mm)					MOULD-DVS 8mm Surface thickness (mm)				
SU%	1	2	3	4	5	1	2	3	4	5	1	2	3	4	5	1	2	3	4	5
1%	43.88	44.46	45.59	45.98	45.98	42.73	43.79	44.78	45.46	45.55	42.0	43.66	44.34	45.77	45.77	42.86	43.97	45.03	45.12	45.3
2%	45.95	47.79	47.79	48.34	48.34	45.77	45.77	46.16	46.16	46.16	45.55	45.81	46.34	46.85	46.92	45.28	45.43	45.73	45.88	46.18
3%	49.89	49.89	50.66	50.66	50.66	46.74	47.37	47.6	48.18	49.11	50.25	50.25	50.25	50.25	50.25	46.45	48.0	48.0	48.1	48.3
4%	57.32	57.32	57.32	57.33	57.33	54.16	54.16	54.16	54.16	54.16	52.54	54.37	58.65	60.8	60.8	53.17	55.16	55.16	55.16	55.16
5%	57.75	57.75	57.75	57.75	57.75	57.39	59.24	59.24	59.24	59.24	52.19	52.27	52.59	52.59	52.59	51.12	51.69	51.69	51.69	51.69

**Table 1B.** Surface Depth Dose for VMAT and IMRT Using VACLOK Immobilisation Device.

VMAT	VACLOK-DVS 1mm Surface thickness (mm)					VACLOK-DVS 2mm Surface thickness (mm)					VACLOK-DVS 3mm Surface thickness (mm)					VACLOK-DVS 4mm Surface thickness (mm)				
SU%	1	2	3	4	5	1	2	3	4	5	1	2	3	4	5	1	2	3	4	5
1%	44.23	46.5	47.63	48.89	49.92	44.81	45.66	47.13	47.77	48.22	44.13	45.69	46.16	47	47.88	43.9	45.16	45.79	46.11	47.64
2%	47.24	48.02	51.1	51.1	51.54	46.2	46.77	47.63	48.63	49.65	45.66	46.57	48.12	48.38	48.64	45.74	46.08	46.93	48.69	49.78
3%	50.95	51.01	51.22	51.35	51.62	48.76	48.76	48.76	50.32	50.37	48.51	49.12	49.92	50.04	50.82	47.75	48.57	49.36	49.36	49.36
4%	50.95	51.01	51.22	51.35	51.62	48.76	48.76	48.76	50.32	50.37	48.51	49.12	49.92	50.04	50.82	47.75	48.57	49.36	49.36	49.36
5%	50.95	51.01	51.22	51.35	51.62	48.76	48.76	48.76	50.32	50.37	48.51	49.12	49.92	50.04	50.82	47.75	48.57	49.36	49.36	49.36
	VACLOK-DVS 5mm Surface thickness (mm)					VACLOK-DVS 6mm Surface thickness (mm)					VACLOK-DVS 7mm Surface thickness (mm)					VACLOK-DVS 8mm Surface thickness (mm)				
SU%	1	2	3	4	5	1	2	3	4	5	1	2	3	4	5	1	2	3	4	5
1%	43.87	44.44	45.05	46.53	47.01	43.85	44.48	44.9	45.74	46.16	44.19	44.39	44.58	45.02	45.33	44.21	44.21	44.21	44.33	44.76
2%	44.83	44.99	46.2	47.03	47.63	44.18	44.99	45.41	45.95	46.47	44.97	45.55	46.67	46.89	47.42	45.1	45.2	45.1	45.3	45.2
3%	46.14	46.82	47.52	49.34	49.34	46.27	46.27	46.88	46.88	47.39	45.31	46.2	46.36	46.38	46.62	45.3	45.3	45.83	45.83	45.83
4%	46.14	46.82	47.52	49.34	49.34	46.27	46.27	46.88	46.88	47.39	45.31	46.2	46.36	46.38	46.62	45.3	45.3	45.83	45.83	45.83
5%	46.14	46.82	47.52	49.34	49.34	46.27	46.27	46.88	46.88	47.39	45.31	46.2	46.36	46.38	46.62	45.3	45.3	45.83	45.83	45.83
IMRT	VACLOK-DVS 1mm Surface thickness (mm)					VACLOK-DVS 2mm Surface thickness (mm)					VACLOK-DVS 3mm Surface thickness (mm)					VACLOK-DVS 4mm Surface thickness (mm)				
SU%	1	2	3	4	5	1	2	3	4	5	1	2	3	4	5	1	2	3	4	5
1%	45.13	47.95	52.52	52.52	53.53	44.8	47.41	51.33	52.5	53.32	45.17	45.52	48.97	50.77	52.21	44.47	45.93	49.03	50.74	50.94
2%	47.8	48.56	54.09	54.09	54.79	46.17	47.91	52.04	53.49	53.49	46.48	47.49	50.79	51.15	52.07	46.68	47.72	48.57	50.21	50.84
3%	48.56	50.03	54.13	54.68	56.98	49.11	49.7	55.0	55.0	55.0	47.24	47.76	49.89	51.81	52.37	48.05	48.4	51.87	53.6	53.67
4%	50.42	53.74	57.86	57.86	57.86	49.0	50.4	51.2	52.32	53.18	47.33	48.05	50.27	52.08	54.18	46.5	49.4	51.86	53.23	53.23
5%	50.42	53.74	57.86	57.86	57.86	49.0	50.4	51.2	52.32	53.18	47.33	48.05	50.27	52.08	54.18	46.5	49.4	51.86	53.23	53.23
	VACLOK-DVS 5mm Surface thickness (mm)					VACLOK-DVS 6mm Surface thickness (mm)					VACLOK-DVS 7mm Surface thickness (mm)					VACLOK-DVS 8mm Surface thickness (mm)				
SU%	1	2	3	4	5	1	2	3	4	5	1	2	3	4	5	1	2	3	4	5
1%	44.36	44.77	46.02	47.22	48.83	44.26	45.47	46.33	47.19	48.05	44.58	45.47	46.08	46.34	46.68	43.83	45.64	47.77	47.79	47.8
2%	45.54	45.54	46.76	47.47	48.11	44.98	47.18	47.74	48.62	49.51	45.39	46.4	47.12	47.84	48.19	45.38	46	46.3	46.3	46.3
3%	48.03	48.03	48.03	48.39	49.09	46.55	46.78	47.32	48.46	49.84	45.16	47.83	49.65	49.65	49.65	44.78	44.78	46.7	46.93	47.4
4%	48.71	48.76	48.84	49.93	49.93	45.65	48.49	48.99	48.99	49.0	46.51	46.51	47.58	49.32	49.32	46.12	46.27	46.96	46.96	47.81
5%	48.71	48.76	48.84	49.93	49.93	45.65	48.49	48.99	48.99	49.0	46.51	46.51	47.58	49.32	49.32	46.12	46.27	46.96	46.96	47.81

**Table 1C.** Surface Depth Dose for VMAT and IMRT Without Immobilisation Device.

VMAT	0PEN-DVS 1mm Surface thickness (mm)					0PEN-DVS 2mm Surface thickness (mm)					0PEN-DVS 3mm Surface thickness (mm)					0PEN-DVS 4mm Surface thickness (mm)				
SU%	1	2	3	4	5	1	2	3	4	5	1	2	3	4	5	1	2	3	4	5
1%	38.45	41.77	42.69	44.38	45.12	37.82	40.46	42.17	43.7	45.08	37.51	39.64	41.85	43.97	44.98	38.0	39.53	41.86	42.99	43.88
2%	39.5	42.62	44.9	48.23	48.24	38.86	42.59	43.7	45.81	46.27	37.91	41.1	43.04	45.07	46.11	37.7	40.59	43.61	44.01	45.1
3%	41.45	44.4	47.66	48.26	48.41	41.27	42.95	46.5	47.41	47.41	38.66	44.61	45.42	45.42	47.16	41.45	42.0	43.02	44.41	46.0
4%	41.45	44.4	47.66	48.26	48.41	41.27	42.95	46.5	47.41	47.41	38.66	44.61	45.42	45.42	47.16	41.45	42.0	43.02	44.41	46.0
5%	41.45	44.4	47.66	48.26	48.41	41.27	42.95	46.5	47.41	47.41	38.66	44.61	45.42	45.42	47.16	41.45	42.0	43.02	44.41	46.0
	0PEN-DVS 5mm Surface thickness (mm)					0PEN-DVS 6mm Surface thickness (mm)					0PEN-DVS 7mm Surface thickness (mm)					0PEN-DVS 8mm Surface thickness (mm)				
SU%	1	2	3	4	5	1	2	3	4	5	1	2	3	4	5	1	2	3	4	5
1%	38.64	39.72	40.94	42.15	43.42	36.69	39.27	40.6	42.74	43.39	37.02	39.23	40.98	42.18	42.99	37.17	39.29	41.01	42.19	43.5
2%	38.68	40.97	42.17	43.53	45.25	37.72	40.68	41.49	43.14	43.93	38.44	40.11	41.67	42.72	44.08	39.1	41.29	42.68	43.68	45.12
3%	39.61	42.52	43.58	45.53	45.55	40.76	44.56	45.99	46.7	47.42	39.04	41.97	42.76	43.19	45.1	39.07	40.58	42.55	43.18	45.34
4%	39.61	42.52	43.58	45.53	45.55	40.76	44.56	45.99	46.7	47.42	39.04	41.97	42.76	43.19	45.1	39.07	40.58	42.55	43.18	45.34
5%	39.61	42.52	43.58	45.53	45.55	40.76	44.56	45.99	46.7	47.42	39.04	41.97	42.76	43.19	45.1	39.07	40.58	42.55	43.18	45.34
IMRT	OPEN-DVS 1mm Surface thickness (mm)					OPEN-DVS 2mm Surface thickness (mm)					OPEN-DVS 3mm Surface thickness (mm)					OPEN-DVS 4mm Surface thickness (mm)				
SU%	1	2	3	4	5	1	2	3	4	5	1	2	3	4	5	1	2	3	4	5
1%	45.12	49.51	52.31	53.58	53.58	44.81	48.74	51.22	51.51	52	43.35	46.68	49.86	51.1	51.66	43.23	46.12	48.64	50.08	50.7
2%	47.24	52.61	52.61	54.95	55.67	45.73	48.93	52.24	54.44	54.44	45.51	48.8	50.45	51.62	52.39	43.63	47.76	49.02	51.76	52.36
3%	48.38	54.55	56.58	56.58	56.58	46.3	53.44	53.44	54.48	55.83	47.88	49.0	52.99	53.61	54.54	49.87	50.28	51.05	53.51	53.51
4%	48.38	54.55	56.58	56.58	56.58	46.3	53.44	53.44	54.48	55.83	47.88	49.0	52.99	53.61	54.54	49.87	50.28	51.05	53.51	53.51
5%	48.38	54.55	56.58	56.58	56.58	46.3	53.44	53.44	54.48	55.83	47.88	49.0	52.99	53.61	54.54	49.87	50.28	51.05	53.51	53.51
	OPEN-DVS 5mm Surface thickness (mm)					OPEN- DVS 6mm Surface thickness (mm)					OPEN-DVS 7mm Surface thickness (mm)					OPEN-DVS 8mm Surface thickness (mm)				
SU%	1	2	3	4	5	1	2	3	4	5	1	2	3	4	5	1	2	3	4	5
1%	42.84	45.71	47.12	48.71	49.78	43.59	46.44	48.09	48.51	48.72	43.43	45.64	46.22	47.26	47.3	43.24	44.55	45.35	45.82	45.91
2%	44.31	46.01	47.92	50.41	51.85	44.59	47.96	48.35	49.14	49.54	44.28	46.85	48.26	49.67	49.67	44.16	45.3	45.42	45.65	46.55
3%	47.03	52.39	52.62	53.62	54.62	45.45	50.37	50.37	50.48	51.0	45.12	48.35	50.71	51.05	51.05	45.49	47.12	47.12	47.12	47.19
4%	47.03	52.39	52.62	53.62	54.62	45.45	50.37	50.37	50.48	51.0	45.12	48.35	50.71	51.05	51.05	45.49	47.12	47.12	47.12	47.19
5%	47.03	52.39	52.62	53.62	54.62	45.45	50.37	50.37	50.48	51.0	45.12	48.35	50.71	51.05	51.05	45.49	47.12	47.12	47.12	47.19

### Statistical Analysis

To evaluate differences between groups, nonparametric statistical analyses were performed using the Kruskal–Walli's test, with a p-value of less than 0.05 considered indicative of statistical significance. Additionally, a two-way analysis of variance (ANOVA) was conducted in R to investigate how varying levels of uncertainty influenced key outcome measures, including effects on the skin, associated devices, and DVS levels, for both treatment plans. To further explore pairwise differences between uncertainty levels, post hoc Tukey tests were applied, with a dose level of 3 mm serving as the reference baseline for comparison. This approach allowed for a detailed assessment of how uncertainty impacts each outcome variable relative to a standardized reference point. For statistical consistency, repeated measurements were performed and evaluated.

## Results

In this study, a total of 40 IMRT and 40 VMAT plans were planned for each of the 3 image sets with varying grid sizes (1 mm −8 mm) and SUs ranging from 1%–5%. The provided data Table 1A,1B &1C appears to be a set values obtained from a study involving different DVS, levels of SU, and different skin thicknesses for three different conditions: with a mould device, with a vaclok device and without an immobilization device for IMRT and VMAT planning.

The results in Table 2 below with Kruskal-Walli's test say significant difference between the groups in VMAT planning with vaclok device and open one, than in IMRT. In most cases, there was a statistically significant difference in skin dose between the different devices (p < 0.05). especially in VMAT planning with open and vac device (<0.05)

**Table 2.** Average Skin Dose and P Value Significance Between Different Devices in Both Plan Using MC Algorithm.

		VMAT					IMRT				
GS	SU	Open	vac	P value	mould	P value	Open	vac	P value	mould	P value
DVS 1mm	1%	41.8±4.7	47.1±4.0	≤0.05	44.5±3.8	0.22	49.4±6.0	49.3±5.9	0.83	46.2±1.8	0.05
	2%	43.9±6.2	49.4±3.0	≤0.05	46.2±2.8	0.88	51.5±6.0	51.3±4.9	0.67	49.1±0.9	0.13
	3%	44.9±4.9	51.3±0.5	≤0.05	47.4±3.2	0.62	52.5±5.8	52.8±6.0	0.43	54.5±0.1	0.25
	4%	44.9±4.9	51.3±0.5	≤0.05	47.4±3.2	0.62	52.5±5.8	54.1±5.3	0.51	59.1±0.0	<0.05
	5%	44.9±4.9	51.3±0.5	≤0.05	47.4±3.2	0.62	52.5±5.8	54.1±5.3	0.51	60.8±0.3	<0.05
DVS 2mm	1%	41.5±5.1	46.5±2.4	≤0.05	44.6±3.5	0.18	48.4±5.1	49.1±6.0	0.83	45.7±1.9	<0.05
	2%	42.6±5.2	47.9±2.4	≤0.05	45.63±.4	0.36	50.1±6.2	49.8±5.2	0.72	48.5±1.7	0.26
	3%	44.3±4.3	49.6±1.1	≤0.05	46.9±1.7	0.52	51.1±6.7	52.1±4.2	0.89	52.2±1.5	0.25
	4%	44.3±4.3	49.6±1.1	≤0.05	46.9±1.7	0.52	51.1±6.7	51.1±3.0	0.29	60.1±4.4	<0.05
	5%	44.3±4.3	49.6±1.1	≤0.05	46.9±1.7	0.52	51.1±6.7	51.1±3.0	0.29	57.9±1.6	<0.05
DVS 3mm	1%	41.2±5.3	46.0±2.7	≤0.05	43.9±3.4	0.23	47.5±5.9	48.7±5.0	0.94	45.1±2.4	0.16
	2%	42.0±5.8	47.2±2.1	≤0.05	45.4±3.1	0.18	49.0±4.9	49.3±4.0	0.67	47.1±2.2	0.52
	3%	42.9± 6.0	49.7±1.6	≤0.05	45.8±2.7	0.62	51.2±4.7	49.8±3.6	0.12	51.7±0.5	0.72
	4%	42.9± 6.0	49.7±1.6	≤0.05	45.8±2.7	0.62	51.2±4.7	50.8±4.8	0.52	53.5±0.3	0.29
	5%	42.9±6.0	49.7±1.6	≤0.05	45.8±2.7	0.62	51.2±4.7	50.8±4.8	0.62	62.6±0.9	<0.05
DVS 4mm	1%	40.9±4.2	45.8±2.6	≤0.05	43.5±2.7	0.18	47.0±5.3	47.7±4.6	0.67	45.1±2.4	0.20
	2%	41.4±5.2	47.8±2.9	≤0.05	44.2±3.2	0.32	48.0±6.2	48.8±2.9	0.83	47.3±1.5	<0.05
	3%	43.7±3.2	48.6±1.1	≤0.05	45.12±.5	0.23	51.7±2.6	50.9±4.0	0.94	50.2±2.20	0.62
	4%	43.7±3.2	48.6±1.1	≤0.05	45.1±2.5	0.23	51.7±2.6	49.9±4.8	0.62	54.1±0.7	<0.05
	5%	43.7±3.2	48.6±1.1	≤0.05	45.1±2.5	0.23	51.7±2.6	49.9±4.8	0.62	58.7±3.4	<0.05
DVS 5mm	1%	41.0±3.4	45.4±2.20	≤0.05	43.7±3.0	0.08	46.3±4.9	46.6±3.2	0.83	44.9±1.5	0.20
	2%	42.0±4.6	46.2±2.0	≤0.05	44.9±3.3	0.07	48.1±5.3	46.8±1.8	0.26	47.1±1.7	0.94
	3%	42.6±4.2	47.7±2.3	≤0.05	44.7±3.5	0.02	50.8±5.4	48.6±0.7	0.01	50.3±0.5	0.48
	4%	42.6±4.2	47.7±2.3	≤0.05	44.7±3.5	0.48	50.8±5.4	49.3±0.9	0.29	57.3±0.1	<0.05
	5%	42.6±4.2	47.7±2.3	≤0.05	44.7±3.5	0.48	50.8±5.4	49.3±0.9	0.28	57.8±0.1	<0.05
DVS 6mm	1%	40.0±4.7	45.0±1.6	≤0.05	43.0±3.1	0.18	46.2±3.6	46.2±2.7	0.44	44.1±2.0	<0.05
	2%	40.8±4.4	45.3±1.6	≤0.05	44.5±2.6	0.10	47.1±3.5	47.2±3.2	0.72	46.0±0.3	0.08
	3%	44.1±4.7	46.8±0.8	0.26	45.3±2.7	0.78	48.2±3.9	48.2±2.3	0.09	47.9±1.7	0.14
	4%	44.1±4.7	46.8±0.8	0.26	45.3±2.7	0.78	48.2±3.9	47.3±2.4	0.28	54.2±0.1	<0.05
	5%	44.1±4.7	46.8±0.8	0.26	45.3±2.7	0.78	48.2±3.9	47.3±2.4	0.28	58.3±1.3	<0.05
DVS 7mm	1%	40.0±4.2	44.8±0.8	≤0.05	42.6±3.3	0.20	45.4±2.7	45.6±1.5	0.83	43.9±2.7	0.09
	2%	41.3±4.0	46.2±1.7	≤0.05	43.7±3.5	0.18	47.0±3.8	46.8±2.0	0.50	46.2±1.0	0.12
	3%	42.1±4.3	46.0±0.9	≤0.05	44.2±3.4	0.23	48.1±4.2	47.4±3.2	0.08	50.3±0	0.83
	4%	42.1±4.3	46.0±0.9	≤0.05	44.2±3.4	0.23	48.1±4.2	47.9±2.0	0.44	56.7±5.8	<0.05
	5%	42.1±4.3	46.0±0.9	≤0.05	44.2±3.4	0.23	48.1±4.2	47.9±2.0	0.43	52.4±0.3	<0.05
DVS 8mm	1%	40.3±4.5	44.5±0.4	≤0.05	42.1±2.8	0.36	44.6±1.9	45.8±2.8	0.26	44.1±1.7	0.36
	2%	42.1±4.3	49.5±0.1	≤0.05	43.6±3.2	0.72	45.4±1.7	45.8±0.7	0.12	45.7±0.6	0.72
	3%	42.2±4.4	45.6±0.4	≤0.05	44.4±1.4	0.23	46.3±1.2	46.1±1.9	0.39	47.4±1.3	0.14
	4%	42.2±4.4	45.6±0.4	≤0.05	44.4±1.4	0.23	46.3±1.2	47.0±1.2	0.62	54.2±1.4	<0.05
	5%	42.2±4.4	45.6±0.4	≤0.05	44.4±1.4	0.23	46.3±1.2	47.0±1.2	0.62	51.4±0.4	<0.05

**Figure 4.**
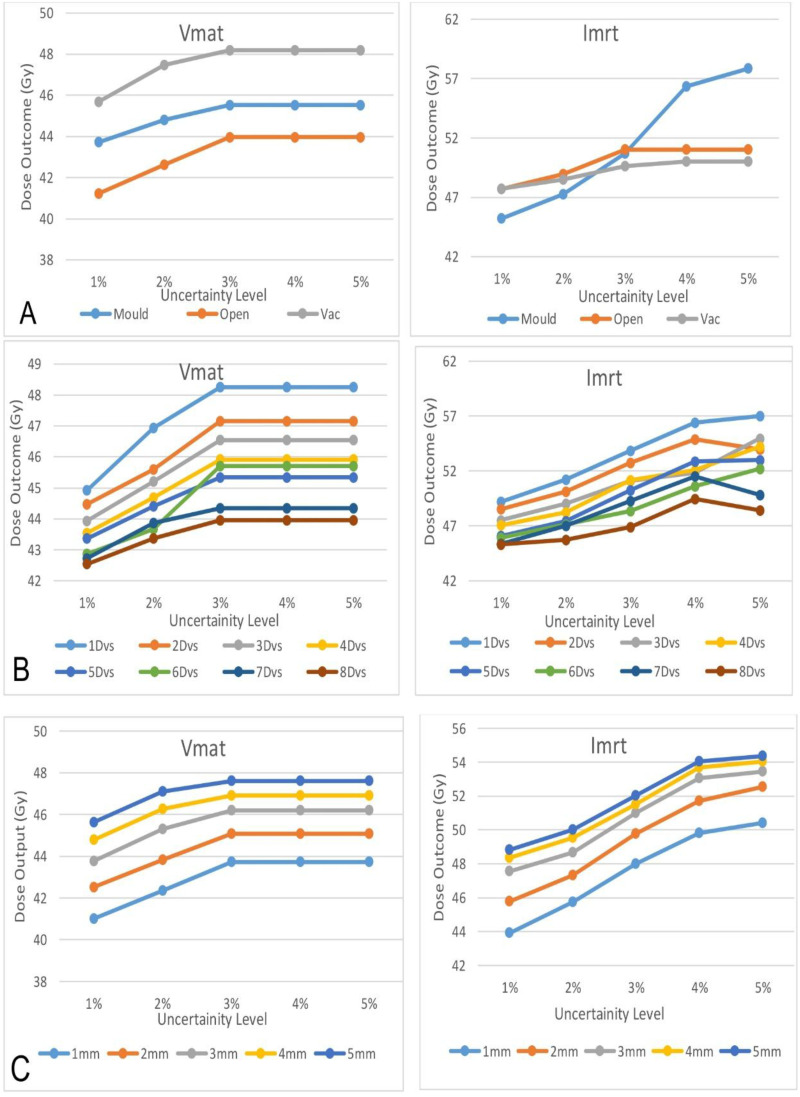
Visualization of (A) Immobilisation devices, (B) Dose Voxel Grid size, and (C) Skin thickness with Uncertainty for IMRT and VMAT Plans.

An R code analysis was conducted via two-way analysis of variance to examine the uncertainty level with respect to the outcome variable effects on the skin, devices and DVS level for both plans. Visualization of (a) Skin Level and Uncertainty level, (b) Treatment devices and Uncertainty level (c) Uncertainty Level and DVS level for IMRT and VMAT are shown in Figure 4 below. Post-hoc Tukey tests are used to compare means between different levels of uncertainty, with a set dose level of 3 mm used as a reference baseline for comparison. Overall, all the factors are statistically significant. It will be simpler to spot trends and interactions because the lines and points for each level will be distinguishable by color and point character.

## Discussion

A wide range of factors influence skin dose, including beam energy, source-to-skin distance, field size, treatment geometry, beam modifying devices, and the use of bolus materials. Beyond these machine and geometry-related factors, the accuracy of skin dose estimation is also closely tied to the DVS and statistical SU settings of the calculation algorithm within the treatment planning system. Understanding how these parameters interact is critical for optimizing treatment accuracy.MC based dose calculation, considered the gold standard for its ability to model particle interactions with high precision. By conducting this rigorous plan study, we aimed to comprehensively gain insight into how MC-based treatment planning, combined with various planning parameters and techniques, influence the dose distribution within the thorax, particularly in the skin layers. This multifactorial approach enables a deeper understanding of the trade-offs involved in planning decisions and highlights the clinical relevance of optimizing calculation settings not only for target coverage but also for minimizing skin toxicity. Several studies have been conducted on the SU and DVS parameters but have generally focused on target coverage or the impact of any one of these parameters. A study performed by Simon K and Martin AE on DVS and SU parameter precision in MC calculations in stereotactic radiotherapy revealed that an SU of 1.55 and DVS of 1 mm are satisfactory for the brain and spine and 2 mm for the lung stereotactic plan.^17^ Another study performed by Jacob R et al on the varying SU in the Monte Carlo algorithm for VMAT planning indicated that increasing the SU per control point exponentially decreased the amount of time required for dose calculations, with minimal observable differences in DVHs and isodose lines.^18^ Palanisamy et al also studied the dosimetric implications of statistical uncertainty in MC dose calculations for VMAT via the Monaco treatment planning system; however, their study was limited to VMAT and varying SU^19^ and not on the surface dose. A study by Asma et al recommended the use of a 3 mm grid size with 1% statistical uncertainty for VMAT head and neck carcinoma plans; however, the work did not address surface dose considerations or compare these parameters across other planning techniques.^20^ To our knowledge, studies comparing the skin dose at depth (1-5mm) with varying SU and DVS with different immobilization and planning techniques have not been performed or analyzed. Rationale for selecting the 1–5 mm skin depth, the most clinically relevant region for assessing acute radiation dermatitis. Since skin toxicity is a major concern in radiotherapy, it is necessary to understand its impact by varying this parameter. Akino et al, Ohtakara K, et al^21,22^ suggested that a smaller grid size can lead to a more accurate dose calculation in the buildup region. Hence, For this study, forty Intensity-Modulated Radiation Therapy and forty Volumetric Modulated Arc Therapy plans were created for each of the three image sets, with grid sizes and Scaling Units varying from 1% to 5%.The vaclok immobilization shows the maximum surface dose in both the planning system, with 51.6 Gy and 57.8 Gy in VMAT, and IMRT, with a 1 mm grid size calculation, followed by a mould device with 49.6 and 61.0 Gy. Typically, when an open device with grid sizes ranging from 1 to 8 is used, the average values fall between 43 and 57. Higher grid sizes and increased levels of uncertainty tend to yield higher average values. Compared with levels 1, 2, and 3, 4 and 5 mm generally have greater average values for different skin levels. When specific devices used in IMRT delivery, such as Vaclok and Mould, are examined, notable dosages are observed at depths within the skin. The Vaclok device shows a high dosage up to a depth of approximately 2–3 mm within the skin layer of 57.8 Gy. On the other hand, the Mould device results in a high dose at depth (1-5mm) at deeper layers, specifically at depths of approximately 4 and 5 mm in the skin dose area, with 49.6 Gy. The average values for the measured data from the Vaclok device typically range between 44 and 57 Gy, depending on the grid size level and uncertainty, which is in line with the trends observed for the Open device. This finding indicates that the choice of immobilization device can significantly impact the measurements. Notably, there was a significant difference in the dose observed with the IMRT plan compared with the VMAT plan, irrespective of the device used. This maximum dose of 57.86 Gy in the IMRT plan may be due to the impact of the beam angle, which was more focused on the target and the surface of the phantom. A study by Gerbi et al also states that a steep angle produces a higher surface dose based on the obliquity factor of the beam used.^23^

In most cases, there was a statistically significant difference in the skin dose between the different devices (p < 0.05). In VMAT planning with open and vac devices (<0.05), for example, open devices had lower skin doses than Vaclok and Mould devices did in several cases. The p values for SU were all less than 0.05, indicating a statistically significant difference between different SU levels. The overall data suggest complex relationships between DVS, SU, skin thickness, and measurement values. Generally, smaller grid sizes and lower SU levels result in lower values, whereas thicker skin tends to result in higher values. Across all grid sizes and skin thicknesses, higher SU levels tend to result in higher values. This implies that a higher SU results in less accurate measurements, alongside a positive correlation between the SU and skin thickness. Caution should be taken when larger grid sizes and higher SU, as this can lead to less precise measurements. The consistency of measurements irrespective of the SU levels indicates that measurement precision may reach a plateau, ceasing to enhance beyond a particular threshold for SU especially after 4%. To ensure accurate skin-dose estimates, an SU level of 2% or lower should be used. However, because the increase in skin dose between SU levels of 3% and 5% is minimal—indicating comparable accuracy—it is more efficient to use SU 5% rather than SU 3% to reduce calculation time. The choice of immobilization device can also have a substantial impact on measurements. The impact of skin thickness should be considered in experimental design, as thicker skin may introduce more variability in the measurements.

The R code analysis via 2-way analysis of variance statistical method is effective for determining how these variables are related to one another. This model also revealed that there was no statistically significant difference between doses 3 and 4 or between doses 3 and 5. There is a change in the outcome of −2.357 units for the 1% uncertainty level compared with the 3% uncertainty level and 2% uncertainty level by −9.338 units, which shows that SU 4% and 5% do not affect the outcome variable compared with those of the 1% and 2% uncertainty levels. If the SU for the plan is set excessively high, the uncertainty will surpass the limit, and the Monaco treatment planning system will disregard the user's input, defaulting to the predefined threshold. Consequently, particle histories will remain unchanged even when the user increases the SU per plan, yielding identical dosimetric outcomes.

The findings from this study demonstrate that planning parameters and immobilization devices exert a measurable influence on surface dose in both IMRT and VMAT techniques when calculated with Monte Carlo algorithms. Variations in DVS and SU revealed consistent trends, larger grid sizes generally produced lower calculated values, while higher SU levels tended to increase values, trade-off between computational precision and statistical stability. Skin thickness was also shown to be an important factor, with greater thicknesses yielding higher measurements, although improvements in accuracy appeared to plateau beyond a certain depth.

Immobilization devices further contributed to these skin dose differences. Among the devices investigated, the vaclok consistently produced the highest measurement values, followed by the thermoplastic mould, with the absence of immobilization the lowest values. The choice of immobilization device is crucial, and the skin dose at depth may introduce more variability. Moreover, the selection of IMRT beam angles and their associated obliquity has a significant influence on skin dose, particularly when a mould immobilisation device is used. The mould material lies directly in the path of the radiation beam and therefore attenuates and perturbs the beam, resulting in inconsistent measured surface dose when compared with other immobilisation devices that are not positioned in the beam path. Researchers should carefully consider these factors in experimental design, as they can significantly affect the results. Further analysis and interpretation should align with the study's specific goals and scientific questions. Jagt et al recently evaluated the influence of Monte Carlo statistical uncertainty on online adaptive radiotherapy workflows and demonstrated that SU settings of 2%–3% were clinically acceptable. Their findings indicated that applying a 2% uncertainty level shortened calculation times, with only minor differences observed in dose–volume parameters.^24^ Ultimately, the choice of algorithm and grid size should align with clinical goals in radiation therapy planning.

Our study has several limitations, such as that the study was based solely on the skin depth associated with the SU and DVS parameters and did not explore the consequences of varying these parameters on OAR. Importantly, treatment planning systems may not accurately measure the dose in the buildup region, particularly for obliquely incident beam angles.^25–28^ Increasing statistical uncertainty in Monte Carlo dose calculations can lead to accumulated calculation errors, particularly in techniques relying on discrete fixed field beamlets such as IMRT, where noise may influence optimization and dose estimates.^14^ Moreover, the dose calculation algorithms have limitations in the buildup region because of the lack of electron equilibrium and incomplete scatter conditions near the skin and air interface. In addition, the study was conducted on an in-house built phantom rather than on patient samples; in the future, this will be considered for further investigations with more immobilization devices.

## Conclusion

Statistical Uncertainty ≤ 2% should be used when accurate skin-dose estimation is required, especially in regions with buildup effects.SU between 3%–5% produces minimal dosimetric difference but substantially reduces computation time; thus SU = 5% is acceptable only when skin dose is not a priority. Grid size of 2–3 mm offers the best balance between accuracy and calculation efficiency. Grid sizes ≥ 5 mm should be avoided when surface dose is of clinical concern, as they underestimate skin dose. Regardless of the devices employed, the skin dose decrease as the grid size increases, and vice versa, with statistical uncertainties. VMAT should be preferred over IMRT when minimizing skin dose, as IMRT consistently produced higher values across all immobilization devices and calculation settings. For clinical sites where skin toxicity is a limiting factor (eg, breast, chest wall), VMAT planning with optimized arc geometry is advisable. The IMRT skin dose at depth (1-5mm) is greater than that of the VMAT plan, irrespective of the device used. Compared with the mould immobilization device and open device, vac-lock immobilization results in the maximum surface dose for both techniques. The results of this study strongly depend on the choice of the beam, its parameters, and the optimization time spent on each plan at the treatment planning station.
